# Supplementary material for: Exploring perceptions and experiences of gender-based violence among women in a refugee camp setting in Uganda—A qualitative study
Source: PLoS One. 2024 Dec 17;19(12):e0314972. doi: 10.1371/journal.pone.0314972 (PMC11651565; doi:10.1371/journal.pone.0314972)
Supplement: Attachment — Submitted filename: PLOS ONE , COMMENTS & SUGGESTION.docx [file pone.0314972.s001.docx]

PLOS ONE

**Manuscript Title**- **Exploring perceptions and experiences of gender-based violence among women in a refugee camp setting in Uganda - a qualitative study**

**Manuscript Number**: PONE-D-24-21429

Dear authors! You are doing a great job. Thank you for conducting this study. Generally, your manuscript has no line number to comment on line by line. So, please try to give them line numbers. There are redundancies in the ideas and words thought out in this manuscript. Please try to reduce repetition of the same ideas and words and rewrite them in a clear and brief manner. **Ethical consideration section**

This study ignored the right of study participants' spouses or partners to let their spouses or partners to participate in this study. Similarly ethical concerns pertaining to refugee camps were not addressed. Who were the data collectors? Were they females or males? Because males are not appropriate for this sensitive issue of data collection.

**Recommendation section**

Your recommendations not in line with your study findings. The recommendation forwarded, “Interventions across various dimensions are therefore warranted to address GBV and gendered power dynamics on multiple levels,” was not specific; it seems general and vague. Please rewrite a specific recommendation based on your study findings.

**Abstract: -** It contains all the scientific content and is written well. But, your recommendation was not appropriate.

**Introduction** correct as “Background”

**Background:** - it was well written and appropriately showed the problem, why this study was important and the gaps seen from previous studies. However, it is lengthy, as if it will be revised and rewritten brief manner. It is better to search for and incorporate previous studies reports about women’s GBV perceptions and experiences into this study background.

Has editorial errors for instances; - 89, 4	26,4	1,5 ??

**Methods**: can be correct as "**Methods and materials."**

For this qualitative study, study setting, design, period, source of population, sampling technique, data collection tools and procedures, and ethical consideration were not separately and clearly written. So, it is better if all the above subtopics under Methods and materials are rewritten in separate subsection and in clear manner.

It was not clear how the study participants were selected and invited from women who were supported by different organizations within single refugee camp

Under the subtopic “**Interview Procedure and Consent**” you stated that” *Eligible participants of the interviews were informed of all aspects of the study by the principal investigator; its purpose, process, risks and benefits, voluntary participation, confidentiality, and all safety and security precautions* *The participants were informed of their right to withdraw from the study at any time without any explanation or consequences.* .” This issue is to address ethical consideration, so it is better to take it to the ethical consideration section.

**Results**: appropriate and well done. But there are sections more focused on socio-economic and health problems that study participants faced at refugee camps. These were not in line with the study title, “Study participants’ perceptions and experiences of GBV”. Your study results should to stick with your study title

**Discussion**

**In my opinion, it was well done and scientifically appropriate.**

**Strengths and limitations**

This study limitation was not well addressed, for instance data collection methods such as focused group discussion and males were not participated to explore their thoughts and perceptions regarding GBV
